# Microbial production of scleroglucan and downstream processing

**DOI:** 10.3389/fmicb.2015.01106

**Published:** 2015-10-15

**Authors:** Natalia A. Castillo, Alejandra L. Valdez, Julia I. Fariña

**Affiliations:** ^1^Laboratorio de Biotecnología Fúngica, Planta Piloto de Procesos Industriales Microbiológicos-CONICETSan Miguel de Tucumán, Argentina; ^2^Cátedra de Micología, Facultad de Bioquímica, Química y Farmacia, Universidad Nacional de TucumánSan Miguel de Tucumán, Argentina; ^3^Cátedra de Microbiología, Facultad de Bioquímica, Química y Farmacia, Universidad Nacional de TucumánSan Miguel de Tucumán, Argentina; ^4^Cátedra de Química Biológica, Facultad de Ciencias Exactas y Naturales, Universidad Nacional de CatamarcaSan Fernando del Valle de Catamarca, Argentina

**Keywords:** scleroglucan, fermentation, bioreactor, optimization, non-conventional substrates, downstream processing

## Abstract

Synthetic petroleum-based polymers and natural plant polymers have the disadvantage of restricted sources, in addition to the non-biodegradability of the former ones. In contrast, eco-sustainable microbial polysaccharides, of low-cost and standardized production, represent an alternative to address this situation. With a strong global market, they attracted worldwide attention because of their novel and unique physico-chemical properties as well as varied industrial applications, and many of them are promptly becoming economically competitive. Scleroglucan, a β-1,3-β-1,6-glucan secreted by *Sclerotium* fungi, exhibits high potential for commercialization and may show different branching frequency, side-chain length, and/or molecular weight depending on the producing strain or culture conditions. Water-solubility, viscosifying ability and wide stability over temperature, pH and salinity make scleroglucan useful for different biotechnological (enhanced oil recovery, food additives, drug delivery, cosmetic and pharmaceutical products, biocompatible materials, etc.), and biomedical (immunoceutical, antitumor, etc.) applications. It can be copiously produced at bioreactor scale under standardized conditions, where a high exopolysaccharide concentration normally governs the process optimization. Operative and nutritional conditions, as well as the incidence of scleroglucan downstream processing will be discussed in this chapter. The relevance of using standardized inocula from selected strains and experiences concerning the intricate scleroglucan scaling-up will be also herein outlined.

## The Discovery of a Challenging Polysaccharide

Around 180-million-tons of polymers are produced per year, which play a relevant role in our modern society. Petroleum-based polymers and polymers of plant origin have the disadvantage of their limited resources in addition to the well-known environmental impact of the former ones. In this context, microbial polysaccharides represent a valuable alternative, with the benefit of lower-cost, standardized and sustainable production, along with high quality. In addition, exopolysaccharides (EPSs) from microbial sources usually exhibit shortened production times (e.g., in a matter of days), the possibility of using industrial wastes, no competition with production lands, and their frequent ease of recovery. Production values in the microbial EPS field can be so varied as 0.0022–86.3 g/L (Donot et al., 2012).

Among microbial polysaccharides, the general term scleroglucan is commonly used to designate a class of EPSs with similar structure, which are mainly -but not exclusively- produced by filamentous fungi of the genus *Sclerotium* (Halleck, 1967). Upon complete hydrolysis, only D-glucose is released from this water-soluble homopolysaccharide. The Pioneering Research Division of the US Army Quartermaster Corps Research and Engineering Centre provided, in Reese and Mandels (1959), the first insights into the scleroglucan structure by enzymatic approaches. Thereafter, in Halleck (1967) patented the scleroglucan basic structure as elucidated by means of enzymatic hydrolysis. This patent also included information about some producing strains, the production processes and the methods of purification thereof (Halleck, 1967). Based on Halleck’s work, Pillsbury Co. (Minneapolis, MN, USA) began scleroglucan commercialization under the name Polytran^®^. Since then, different companies entered into the scleroglucan market under different trademarks (Clearogel, Polytetran, Polytran FS, Sclerogum, and Actigum; Coviello et al., 2005).

## Scleroglucan Chemical Structure and Conformational Features

**FIGURE 1:**
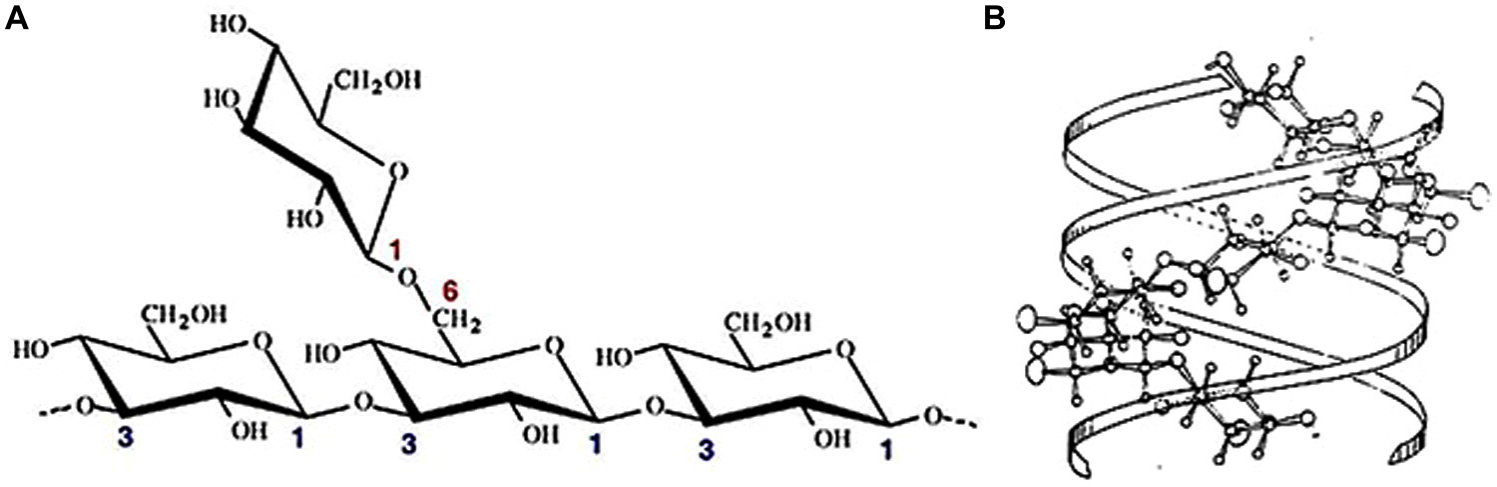
**(A)** β-(1,3)-β-(1,6) glucan structure exhibiting the (3:1) side branching ratio of scleroglucan (Martin et al., 2007). **(B)** Tridimensional conformation of scleroglucan triplex. Projection perpendicular to the fiber axis (Crescenzi et al., 1988).

Scleroglucan is a high molecular weight (MW), non-ionic branched glucan. It consists in a backbone of (1,3)-β-linked D-glucopyranosyl residues bearing a single (1,6)-β-linked D-glucopyranosyl unit every three sugar residues of the main chain (Rinaudo and Vincendon, 1982; Fariña, 1997). The structure of this repetitive unit determines a degree of branching (DB) around 0.33 (**Figure 1**). Besides being a common feature among most biologically active β-(1,3)-glucans (Rinaudo and Vincendon, 1982; Bohn and BeMiller, 1995; Kim et al., 2000), this high branching frequency would also be responsible of the great water solubility of this polysaccharide. When dissolved in water at room temperature and low concentrations of alkali, usually below 0.15 M NaOH, it can be assumed that scleroglucan adopts a highly ordered, rigid, triple helical tertiary structure (**Figure 1**). Under this macromolecular conformation, protruding (1,6)- β-glycosidic side branching prevents the intermolecular approach by extensive H-bonding, which otherwise would lead to aggregated forms and precipitation (Fariña et al., 2001, 2009; Laroche and Michaud, 2007). Meanwhile, interstrand hydrogen bonding at the center of the triplex stabilizes the macromolecular structure (Atkins and Parker, 1968; Bluhm et al., 1982; Sletmoen et al., 2009). However, at higher NaOH concentrations, where drastic changes in viscosity are commonly observed, the triple-strand helices probably undergo the ionization of hydroxyl groups which thus disrupts hydrogen bonds and prompts the subsequent polysaccharide denaturation (Fariña et al., 2001; Viñarta et al., 2013a).

To deepen into the knowledge of this denaturation–renaturation process, Virgili Alemán (2011) monitored, by fluorescence resonance energy transfer (FRET) spectroscopy, the conformational changes of scleroglucan triplexes when exposed to different NaOH concentrations. This study revealed that triple helix denaturation would take place by a partial opening mechanism, with a degree of opening related to both the NaOH concentration and the EPS conformational features (degree of expansion) of the triplexes.

The length of polymer chain, and therefore the molecular mass of scleroglucan, may differ depending on the microbial strain, fermentation process (culture media, fermentation time, etc.) and the recovery method used, with an average MW ranging from 1.3–3.2 × 10^5^ to 0.3–6.0 × 10^6^ Da (Rodgers, 1973; Fariña et al., 2001). Within this range, *Sclerotium rolfsii* ATCC 201126 scleroglucan exhibits an average MW of about 5.2 × 10^6^ Da for the triplex and 1.6–1.7 × 10^6^ Da for the random coil, in association to high intrinsic viscosities ([η] = 9510–9610 mL/g, for the triplex in water; Fariña et al., 2001). With reference to the degree of polymerization (DP), the reported values are variable from 110 for *Sclerotium glucanicum* scleroglucan (Bielecki and Galas, 1991), 800 for a commercial scleroglucan (Bluhm et al., 1982), 500–1600 for related glucans (Sandford, 1979), 2400–2500 for *S. rolfsii* ATCC 201126 scleroglucan (Fariña et al., 2009) and up to 5600 for another cited scleroglucan (Rice et al., 2004).

A tendency to adopt a highly ordered, triple-helical conformation and semi-rigid structure in neutral aqueous solution, in association to high DP and MW (∼5 × 10^6^ Da) values, may account for the marked viscosifying ability, outstanding rheological behavior and the emerging scleroglucan successful applications (Yanaki et al., 1981; Brigand, 1993; Falch et al., 2000; Fariña et al., 2001; Viñarta et al., 2006, 2007; Giavasis, 2014).

## A Brief Pane on Scleroglucan Properties and Applications

Scleroglucan exhibits a range of distinctive physico-chemical properties that provide an advantage to itself over other polysaccharides, especially for the development of certain products and processes. Nevertheless, slight to great variations of these properties may be seen depending on the producing strain, the polymer production process and the downstream processing, facts that might modify the MW, DP, DB, conformational parameters, and/or the polymer purity grade, and so will determine its final potential applications. For instance, we reported that low concentrations (e.g., 2 g/L) of pure (∼90–98% EPS) *S. rolfsii* ATCC 201126 scleroglucan in water are able to yield highly viscous solutions with non-Newtonian, non-thixotropic and pseudoplastic behavior (Fariña et al., 2001, 2009; Viñarta et al., 2007).

Solutions of scleroglucan are notably stable over temperature up to 100–120°C, and within a broad range of pH (1–13). Additionally, the EPS neutral nature allows keeping pseudoplasticity even in the presence of a variety of salts, such as NaCl, KCl, CaCl_2_, MgCl_2_, and MnCl_2_ (Fariña et al., 2001; Viñarta et al., 2013a). In contrast, slightly refined solutions (2 g/L) of commercial scleroglucans and crude polymer isolates from fermentation broths produce lower viscosity solutions with a lesser ability to retain stable rheological features when exposed to alkali, high temperatures, or salts (Wang and McNeil, 1996; Viñarta et al., 2007, 2013a). Differences depending on the EPS purity grade were also found between Biopolymer CS6 (60–70% scleroglucan) and Biopolymer CS11 (85–90%; Survase et al., 2007a). Based on these and other outstanding scleroglucan properties, a wide spectrum of biotechnological and industrial applications has been proposed and evaluated up to date (**Table 1**).

**Table 1:** Summary of the main scleroglucan applications, according to its refined grade and its physico-chemical, biological, and biotechnological properties.

Scleroglucan source	Highlighted scleroglucan properties	Proposed applications	Reference
Actigum CS-11^∗^ (formerly known as Polytran^®^from Ceca S.A., France)	• Water solubility • Viscosifying activity • Salts tolerance	Scleroglucan aqueous fluid, used for petroleum recovery	Doster et al., 1984a,b
*Sclerotium rolfsii* ATCC 15206 Actigum CS-11^∗^ and Actigum CS6^∗∗^, by Sanofi Bio Industries, France	• Thickening power • High carrying capacity and lubricating power	Scleroglucan gels for enhanced oil recovery (EOR)	Holzwarth, 1984; Donche et al., 1994; Pirri et al., 1996
Actigum CS-11^∗^ and CS-6^∗∗^, by Sanofi Bio Industries, France	• Viscosifying ability • Rheological stability vs. temperature, salinity, and pH	Viscosity control of a bituminous binder for road repair and construction, soil stabilization, and sealing in civil engineering	Chaverot et al., 2001
Polytran^®^, by Pillsbury Company, USA	• Stimulation of murine macrophage activity • Increase in murine macrophage tumor cytotoxicity • Enhancement of murine bone marrow proliferation • Increase in survival of carcinoma-challenged mice • Increase in mice resistance against pathogenic bacteria and virus, w/o toxicity or hepatomegaly	Immune stimulating	Pretus et al., 1991
Not specified	• Apt matrix for controlled drug delivery • Drug protection at gastric level • Bioadhesive properties • Biodegradability	Edible films and tablets for nutraceuticals and pharmaceuticals Capsule granulates for controlled release of active substances	Lovrecich and Riccioni, 1991, 1993
Actigum CS11^∗^, by Mero Rousselot - Satia, France	• Biocompatibility • Thermal and chemical stability		Grassi et al., 1996; Coviello et al., 1999
Not specified	• Antitumour, antiviral, and antibacterial activity (native or derivatized)	Drugs, vaccines, and immuno-potentiators (combinable with chemotherapy)	Jong and Donovick, 1989; Giavasis, 2014
Scleroglucan, by Sanofi Bio Industries, France	• Hypocholesterolemic, hypolipidemic, and hypoglycemic	Nutraceuticals	Mastromarino et al., 1997
Scleroglucan purified from fermentation broth, provided by Statoil/Norferm	• Stabilizer-texturizer at low pH and high temperature • Edible-film forming properties	Functional foods Low-calorie foods (since non-digestible)	Falch et al., 2000
Actigum CS6^∗∗^ and CS11^∗^, by Sanofi Bio Industries, France			Vaussard et al., 1997
Actigum CS-11^∗^, by Sanofi Bio Industries, France Amigel, by Alban Muller International	• Homogeneity and thickening enhancer • Softness developer	Component for washing keratinous materials (e.g., shampoo, shower gel, conditioner)	Dubief, 1996; Dubief and Cauwet, 2000
Actigum CS-11^∗^, by Satia, France	• Remains elastic at saliva viscosity (1.5–3 mPa.s), at a physiological pH (5–9), and at physiological ionic strength (15–80 mM)	Saliva substitution agent Artificial tear water Mouth rinse Toothpaste	Van Nieuw Amerongen et al., 1999
Actigum CS-11^∗^ and CS-6^∗∗^, by Sanofi Bio Industries, France	• Viscosifying agent of polyol base solvents • Long term stability at high temperatures and against metal ion contaminants	Rheological modifier for thermal insulation fluids	Skaggs and Swazey, 1999
*S. rolfsii* ATCC 15205	• Forms flexible, insoluble in water films when dried, but swell readily • Dispersing agent • Smoothing agent • Lubricating agent • Emulsifier or co-emulsifier agent • Improvement of fixing of dyes or UV-absorbers in shampoo/conditioners • Skin anti-inflammatory effect	Useful as active and/or excipient ingredient in cosmetic formulations (shampoo, conditioner, after-sun preparations, skin care compositions) and ophthalmological preparations	Maier et al., 2000, 2002
Not specified	• Compatible thickening agent	Component of a cosmetic mixture for the oxidation tinting of keratin fibers	Lang and Cotteret, 2003
BIOVIS, by SKW, Germany	• Suspending agent	Formulation of an aqueous storable cement used for cementing an area of a borehole	Fanguy et al., 2006
*S. rolfsii* ATCC 201126	• Retrogradation preserving agent in cooked starch pastes • Synergic rheological improvement of starch-based pastes (in water and milk)	Food stabilizer	Viñarta et al., 2006
	• Gel and film forming properties • Particulate suspending properties • Emulsifying activity	Drug delivery Paper, painting, ceramic, cosmetic, food and pharmaceutical industries Bioremediation, agriculture, and detergents	Viñarta et al., 2007
Not specified	• Gelling and viscosifying properties • Resistance to degradation, even at high temperatures and after 500 days in seawater	Industrial and oil field operations where acidizing procedures are applied	Welton et al., 2009a,b
Not specified	• High flocculant capacity • Fast settling rate	Addition to liquor of a Bayer process fluid stream to improve the recovery of alumina trihydrate	Chester et al., 2012a,b, 2013

*^∗^Biopolymer CS-11 is a refined product and has a content of 85–90% polysaccharide.*

*^∗∗^Biopolymer CS-6 contains 60–75% scleroglucan.*

Scleroglucan as triplex exhibits the tendency to form thermo-reversible gels at low temperatures (close to 7°C), due to a weakly interacting triple-helix cross-linking mechanism (Bluhm et al., 1982; Biver et al., 1986). On the other hand, mimicking the behavior of other closely related β-(1-3)-D-glucans, scleroglucan triple helices can also be affected by denaturing conditions (e.g., pH ≥ 13), where destabilization of the interstrand H-bonding leads to the dissociation into single stranded random coils (Deslandes et al., 1980; Norisuye et al., 1980; Bluhm et al., 1982; Yanaki and Norisuye, 1983; Ensley et al., 1994). Denaturation of triplexes may occur in alkaline solutions (≥0.25 M NaOH), in dimethylsulfoxide (DMSO; water weight fraction, WH < 0.13), or by increasing the temperature above the triplex melting temperature (T_m_ ≅ 135°C; Fariña et al., 2001; Sletmoen et al., 2009; Viñarta et al., 2013a). Typically, denatured solutions show much lower or nil viscosity as compared to the triplex-containing solutions. Nevertheless, under certain conditions, if denatured samples are taken to conditions that favor the restoration of the triple helical structure, circular structures might be observed by ultramicroscopy techniques among the “renatured triplexes” (Stokke et al., 1991, 1993; Sletmoen et al., 2009).

With regard to the scleroglucan biological properties, it was reported that its administration by diverse routes in rats and dogs did not induce toxicity, tissue pathology, or blood abnormalities. Neither eye nor skin irritation was detected in pigs, rabbits, and humans. Furthermore, scleroglucan role as an immune stimulant and a non-digestible dietary fiber for humans has been reported (Rodgers, 1973; Rapp, 1989; Giavasis, 2014). A wide range of physico-chemical, nutritional, and biological properties have been extensively described in the literature, and certainly are worth to mention. Relevant activities for health involve hypocholesterolemic, hypoglycemic, health-promoting effects, antioxidant and anti-obesity properties, many of them applicable for developing functional foods or nutraceuticals (Giavasis, 2014). A general overview of relevant polymer features and their actual or potential implications are depicted in **Table 1**.

## Reviewing the Knowledge and Advances on Scleroglucan Production

To date, all scleroglucan production processes take place with a selected producing strain and under submerged aerobic conditions. This process is generally carried out in stirred-tank reactors using a sterile medium under aseptic management of the culture. Scleroglucan synthesis proceeds along with mycelial growth, so that the culture broth develops with time a gel-like consistency (Rodgers, 1973). A sharp drop in pH (∼2–2.5) is normally observed during the first 12–24 h of cultivation, mainly due to the accumulation of oxalic acid (Maxwell and Bateman, 1968; Fariña et al., 1998; Lee, 1998).

As aforementioned, changes in culture medium composition, process parameters or even the downstream processing may lead to dissimilar scleroglucan recovery and quality, with eventual variations in its chemical, physical, and/or biological properties. Therefore, in order to obtain high yields of a consistent polymer, it becomes essential to standardize a large-scale production process with a given strain under controlled conditions (Fariña et al., 1998; Survase et al., 2007a; Fazenda et al., 2008; Seviour et al., 2011a). A quite relevant step consists in selecting an appropriate producing strain, whose preservation procedure should be assessed and standardized, and its production ability must be monitored over time (Fariña et al., 1996; Survase et al., 2006; Schmid, 2008).

The nutritional requirements and culture conditions are commonly evaluated at minor scale (i.e., shake flasks) at the beginning of optimization, in order to maximize scleroglucan production and simultaneously reduce the accumulation of unwanted by-products, such as oxalic acid (Fariña et al., 1998; Schilling et al., 2000; Valdez, 2013). Following these essential studies, the scaling-up to bioreactor becomes a critical but difficult step, and this issue will be discussed later.

**FIGURE 2:**
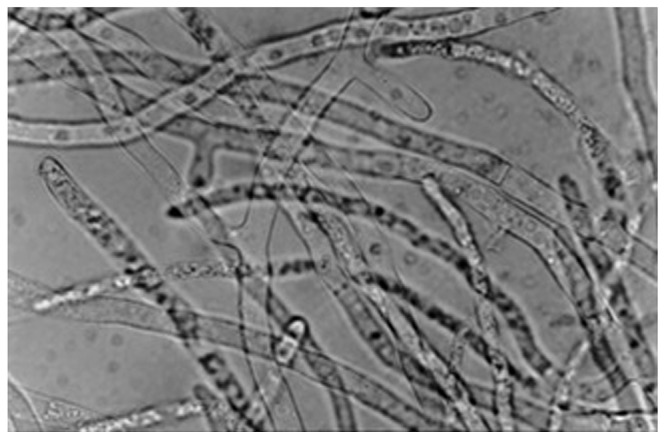
**Microscopic (40×) observation of *Sclerotium rolfsii* ATCC 201126 mycelium after growth for 3 days at 30°C in liquid PM_20_ (with 20 g/L sucrose, Fariña et al., 1998).** Mycelium was disaggregated in 10% w/v KOH.

From our research on scleroglucan production during the last two decades, *S. rolfsii* ATCC 201126 (**Figure 2**) became the selected strain to obtain vast amounts of EPS. This usually involved batch processes at different working scales and/or with varying fermentation strategies. This strain was isolated in the field as a phytopathogen from rotten red pepper. Based on our screening, it was later deposited and cataloged in the ATCC because of its marked ability to excrete biopolymer. There are several aspects that must be considered in order to achieve stable and maximum EPS productivity levels, many of them which are shared in common with other microbial polysaccharide production processes, and these will be herein revisited.

### Strain Preservation

**FIGURE 3:**
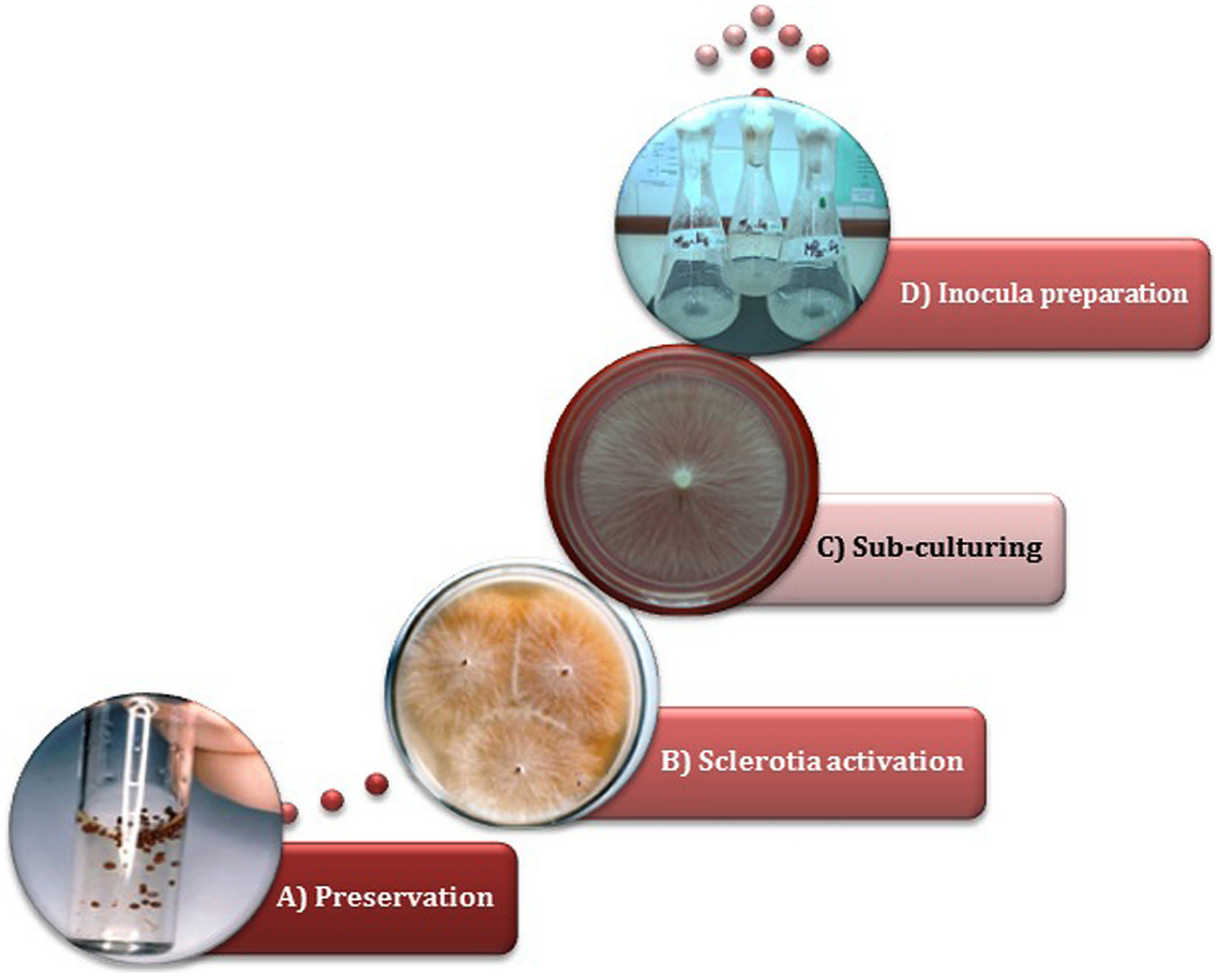
**Inocula preparation starting from sclerotia of *S. rolfsii* ATCC 201126.** Sequence order:→ **(A)** Sclerotia preserved in sterile distilled water →**(B)** Sclerotia germinated in Czapek malt agar →**(C)** Sub-culturing in PM_20_ agar →**(D)** Cultivation in PM_20_ liquid medium, at 220 rpm and 30°C (Fariña et al., 1998).

Although it may seem a minor issue, an adequate strain preservation technique is undoubtedly recognized as a crucial strategy in order to assure long-term viability as well as the maintenance of fungal properties (Smith, 1988). In the first reports, scleroglucan-producing strain conservation was performed by monthly transfers either on PDA or PDY slants (Christias and Lockwood, 1973; Griffith and Compere, 1978; Punja et al., 1982; Pilz et al., 1991). While this is a quite common methodology, an alternatively described technique consists in the preservation of mycelium in sterile distilled water (also known as Castellani’s method; American Type Culture Collection, 1982). These above and other methods were tested with different *S. rolfsii* strains isolated from nature (Fariña et al., 1996). Early reports revealed that periodic sub-culturing on different culture media, followed by preservation at low temperature (4–7°C), may lead to a lack of viability and a critical decrease in scleroglucan synthesizing capacity. Conversely, preservation as ‘sclerotia’ (the resistance structures of the non-sporogenic *S. rolfsii*) in sterile distilled water at 4°C or even at room temperature (**Figure 3**), allowed the retention of the glucanogenic ability at similar and even higher levels than those observed for the abovementioned methods, and even after years of preservation (Fariña et al., 1996).

### Inoculum Standardization

The success of scleroglucan production at bioreactor scale also closely depends on the inoculum proportion and its quality, which must be standardized (van Wollingen and Seviour, 1986). It is known that the preparation of a standard inoculum is usually problematic since the genus *Sclerotium* does not produce spores, and because of the non-homogeneous nature of mycelial suspensions. However, *Sclerotium* genus has the ability of forming resistance structures called sclerotia, which can be thereafter used for strain activation and inocula preparation (Fariña et al., 1996; Survase et al., 2006). It has been demonstrated that activation of water-stored sclerotia followed by sub-culturing in liquid production medium (PM) allows the proper preparation of relatively homogeneous suspensions for inoculation (**Figure 3**), being possible to achieve scleroglucan concentrations similar to those obtained with the strain periodically activated (Fariña et al., 1996).

Another approach that greatly contributes to the preparation of homogeneous inocula is the inclusion of a homogenization step of mycelium-covered agar plugs, suspended in the appropriate volume of culture medium. Standardized proportions should then be used to assure reproducibility, and the inoculum preparation can be safely performed with the aid of a hand blender at controlled speed and for a given time, under aseptic conditions (Fariña et al., 1998).

### Cultivation Conditions

As for any other microbial process, scleroglucan production requires some specific culture conditions which become critical in order to achieve maximum productivity. These not only involve nutritional requirements of the producing strain but also operative conditions such as pH, temperature, aeration, agitation, foam control and inoculum size, among the most representative ones. For scleroglucan production with *S. rolfsii* ATCC 201126, many of these conditions were first experimentally adjusted at flask scale and then scaled-up to bioreactor. Additionally, other groups working on scleroglucan production have also evaluated most of these parameters and showed agreement or not with our findings with *S. rolfsii* ATCC 201126. These results will be subsequently discussed.

#### Nutritional Requirements

Several reports state that broth concentrations of fungal EPSs can be both affected by the nature of carbon and nitrogen sources, as well as by their initial concentration in culture broth (Deshpande et al., 1992; Seviour et al., 2011b). It was also described that highest amounts of biomass do not always lead to optimal EPS production (Giavasis et al., 2005). In the case of *S. rolfsii* ATCC 201126, as also found for other scleroglucans, a high carbon to nitrogen ratio in culture medium would be required to enhance EPS biosynthesis (Fariña et al., 1998; Survase et al., 2007a). Additionally, concerning the nature of nutritional sources, higher polymer concentrations would be associated to the preferential use of sucrose as C-source and NaNO_3_ as N-source. On the other hand, N-sources such as (NH_4_)_2_SO_4_ and other NH_3_-based N-sources led to a significant decline in scleroglucan production, a fact likely related to a negative metabolic regulation on the EPS biosynthetic machinery by ammonium (Fariña et al., 1998, 1999). Similar findings were observed for the EPS formation by *Phoma herbarum* CCFEE 5080 (Selbmann et al., 2002b). As also described for pullulan production (by *Aureobasidium pullulans* ATCC 9348), the initiation of scleroglucan production by *S. rolfsii* ATCC 201126 did not coincide with nitrate exhaustion (Campbell et al., 2003). In addition, carbohydrates other than sucrose and glucose, or eventually non-conventional substrates, could alternatively be used as C-sources for scleroglucan production (Wang and McNeil, 1996; Selbmann et al., 2002a; Survase et al., 2007b; Fosmer et al., 2010; Schmid et al., 2011; Valdez, 2013).

Among culture medium characteristics, the C-source concentration has normally exhibited a remarkable influence on scleroglucan production (Fariña et al., 1998; Survase et al., 2006). As emphasized above, a high C:N ratio usually favors EPS production, and different reasons were speculated for this observation. One rational explanation would be the preferential use of the C-source to produce a carbonaceous product (polysaccharide) with lower osmotic effects than the original sugar substrate (sucrose), which would be available for growth under future starvation conditions (Nelson et al., 2008). On the other hand, an increase in scleroglucan production growing under high-osmotic pressure conditions (e.g., 150 g/L sucrose) could reasonably be part of an osmoregulation mechanism (Fariña et al., 1998), as discussed below (see Effect of Other Factors). Scleroglucan production has been mentioned to occur in parallel with cell rescue processes and alternative energy-generating pathways, which may explain the EPS overproduction as a mechanism for survival under anoxic or other stressing conditions (Schmid et al., 2011).

In the case of *S. rolfsii* ATCC 201126, a 7.5-times increase in carbon concentration as compared to the basal medium (150 g/L vs. 20 g/L of sucrose, respectively), led to a fivefold increase in EPS concentration after 72 h of cultivation at shake-flask scale, with a similar behavior at fermenter scale (Fariña et al., 1998, 2001). The detection of glucanases (β-1,3-glucanase, β-1,6-glucanase, and β-glucosidase) under C-source limiting conditions along with the ability to degrade scleroglucan was early reported for *S. glucanicum* and *S. rolfsii* (Rapp, 1989; Martin et al., 2007). In culture broths of *S. rolfsii* ATCC 201126, reduced extracellular β-glucanase activity could be detected at the end of cultivation with low sucrose concentrations (e.g., 20 g/L sucrose for inoculum preparation). However, after transferring the culture to the fermenter with a high-sucrose PM (MOPT with 150 g/L), glucanases became undetectable. The measured high residual sucrose concentrations, even at the end of cultivation in MOPT (ca. 80–100 g/L), may repress β-glucanase activity (Rau, 2004). Nevertheless, high titers of EPS would be more likely related to an osmotically induced β-glucan synthesis than to this glucanase catabolic repression (Fariña et al., 1998, 2009; see Effect of Other Factors). In addition, previous reports on fungal glucan synthetase activity demonstrated that the concentration of sucrose proved to be crucial for enzyme stability at 30°C (Leal et al., 1984; Finkelman and Vardanis, 1986).

Culture media with concentrations of glucose or sucrose (30–35 g/L) lower than that one (150 g/L) being optimal for *S. rolfsii* ATCC 201126 have been reported by different authors, with a maximum production of 8.5–10 g EPS/L (Wang and McNeil, 1995d; Schilling et al., 2000). Nevertheless, the C-source requirements seem to be strain-specific. In this sense, it has been found for example that growth of *S. glucanicum* is completely inhibited by sucrose concentrations above 45 g/L, which further limits the scleroglucan production (Wang and McNeil, 1994). On the other hand, similar to the effects of high sucrose concentrations in *S. rolfsii* ATCC 201126 (Fariña et al., 1998), Survase et al. (2006) reported a maximum production of 16.5 g EPS/L with a sucrose concentration of 80 g/L.

Other components of culture medium exhibited lesser influence on scleroglucan production. In *S. rolfsii* ATCC 201126, a change in phosphate concentration from 1.3 to 2 g/L led to higher scleroglucan concentrations. Meanwhile, additional constituents such as L-threonine and ascorbic acid significantly decreased scleroglucan production (Fariña et al., 1998). With regard to nitrogen and phosphate sources, Taurhesia and McNeil (1994a) found that higher titers of scleroglucan from *S. glucanicum* were produced in a P-limited medium (18.9 g/L of scleroglucan) than in a N-limited medium (11.4 g/L EPS). High scleroglucan concentrations have also been commonly supported by the presence of yeast extract and casein hydrolysates in culture medium (Taurhesia and McNeil, 1994a).

To conclude, with regard to the scleroglucan production by *S. rolfsii*, there seems to be much agreement in the culture medium composition over the latest years, using optimized culture medium formulations similar to MOPT, which contain a high C:N ratio with sucrose as C-source (e.g., 150 g/L), NaNO_3_ as N-source (in the order of 2.25 g/L), K_2_HPO_4_⋅3H_2_O as P-source (∼2 g/L) and other minor components (in g/L): KCl, 0.5; MgSO_4_⋅7H_2_O, 0.5; yeast extract, 1; citric acid⋅H_2_O, 0.7; FeSO_4_⋅7H_2_O, 0.05 (initial pH adjusted to 4.5). Cultivation of *S. rolfsii* ATCC 201126 at eight L-fermenter scale by using this culture medium led to the highest EPS production kinetic parameters (i.e., 26 g scleroglucan/L, theoretical yield factor, *Y_p/c_* = 0.68 and volumetric productivity, *Pr* = 0.542 g/L⋅h) at 48 h of fermentation (Fariña et al., 1998).

#### Culture Conditions

The control and maintenance (or modulation) of operative parameters becomes fundamental for a process optimization. Some typical aspects for the particular case of scleroglucan production will be discussed below. Along the scleroglucan research history, many researchers and engineers have ventured different alternatives in a continuous effort to improve EPS production and purification, and some of these will be herein revisited.

##### Effect of temperature

This parameter typically affects both culture growth and polysaccharide production. However, it has been reported that in batch cultures, maximum EPS biosynthesis is achieved at temperatures somewhat lower than the one for optimal growth rate. When the organism growth rate is decreased by reducing cultivation temperature, this may increase the availability of isoprenoid lipid carrier for non-growth functions, thus stimulating polysaccharide production (Wang and McNeil, 1996; Fosmer and Gibbons, 2011). Optimal biomass production usually occurs at temperatures above 28°C, while “optimum” temperature for scleroglucan formation was found to be ∼28°C (Giavasis et al., 2005). Instead, below 28°C, by-product (oxalic acid) formation is gradually increased, so that at 20°C acid production may exceed biomass and EPS biosynthesis (Wang and McNeil, 1995b). In the case of *S. rolfsii* ATCC 201126, the production process is commonly carried out at 30°C, obtaining optimal EPS yields.

##### Effect of pH

This factor frequently influences the microorganism physiology by affecting both nutrient solubility and uptake, enzyme activity, cell membrane morphology, by-product formation, redox reactions, etc. (Giavasis et al., 2005). As described for temperature, the appropriate pH for maximum polysaccharide production can differ from that for optimal growth. Kang and Cottrell (1979), pointed out that the optimum pH for fungal polysaccharide synthesis usually lies between 4.0 and 5.5.

First studies of pH effects on scleroglucan production by *S. rolfsii* ATCC 201126 were performed at shake flask scale where, after initial adjustment, pH was left uncontrolled. Comparison of such results to those obtained in stirred-tank reactors is not very different, as most of the cultivations up to date gave the best EPS values when pH was not fixed to a given set-point. It naturally reaches values of 2–2.5 at around 18–24 h of fermentation with a slow and slight increase afterward (Fariña, 1997; Fariña et al., 1998; Valdez, 2013). However, other authors recommended the control of pH during scleroglucan fermentation with *S. glucanicum* NRRL 3006 in order to achieve higher polysaccharide titers (Wang and McNeil, 1995c). As the automatic pH control implies extra costs and further handling of the batch, this aspect will require a proper examination depending on the species and/or the strain to be employed.

##### Effect of dissolved oxygen (DO)

For aerobic organisms, particularly for fungi, oxygen plays a vital role in many aspects of cellular metabolism. Changes in dissolved oxygen often impact respiration rate, enzymatic synthesis and activity, formation of metabolic products, etc. (Forage et al., 1985). Rau et al. (1992) investigated the effect of DO on polymer biosynthesis by *S. glucanicum*. They reported that a high oxygen supply resulted in increased cell growth rate along with decreased scleroglucan production. In contrast, when oxygen partial pressure in the liquid phase was almost reduced to zero, the fungal response was a limited growth but the specific stimulation of scleroglucan formation (Rau et al., 1992; Schilling et al., 1999). Wang and McNeil (1995a) also suggested that, when oxygen becomes limiting, growth undergoes a severe restriction for the C usage, which in turns can be derived toward different metabolic activities such as polymer and/or oxalate production.

The effect of limiting DO tension on the stimulation of EPS production may be difficult to understand; biopolymer synthesis is an energy-demanding process and the generation rate of energy in aerobic microorganisms always relates to the oxygen supply. A reduction in culture DO, however, may disturb fungal morphology, broth rheology or C assimilation, in a favorable way for EPS biosynthesis (Olsvik and Kristiansen, 1994). Nevertheless, since EPS production has been found to start before the DO levels reach limiting values, it becomes unlikely that oxygen limitation triggers scleroglucan biosynthesis by itself (Wang and McNeil, 1995a).

A previous report suggested that at high respiration rates (high DO tension) more carbon would be converted into carbon dioxide, thus leaving less available C-source for scleroglucan production (Sutherland, 1977). Reaffirming this assumption, Wang and McNeil proposed that low DO levels would lead to restricted *Sclerotium* fungal growth (which is an aerobic process) and consequently, more C-source could be driven to scleroglucan biosynthesis. This is in agreement with the statement that EPS production and cell growth are alternative fates of the C-source. Therefore, it sounds very realistic the rationale that, under certain conditions, the improved availability of C is governing polysaccharide production (Wang and McNeil, 1995a). Moreover, low DO levels might decrease by-product (oxalate) formation by repressing the synthetic enzyme glycolate oxidase (Schilling et al., 2000), thus reducing the diversification of the C-source to undesirable products, and simultaneously favoring the C-flow toward β-glucan production.

##### Effect of aeration and agitation rates

Optimizing aeration and agitation rates represents a critical tool to control cell growth and scleroglucan production. These parameters are related to an adequate culture mixing and the sufficient mass and heat transfer rate, which can therefore increase the transference of nutrients and oxygen from liquid medium to the cells, and also modulate the rate of metabolite release from cells to the surroundings, this latter including biopolymers, by-products, and carbon dioxide (Giavasis et al., 2005).

As aforementioned, EPS production is characterized by the development of a very viscous culture broth that commonly exhibits Non-Newtonian, pseudoplastic rheological behavior, emphasized by the production of high biomass concentrations. Furthermore, at moderate to low stirring rates, the fungus forms mycelial pellets. These both phenomena result in the development of a very heterogeneous culture, where nutrient and oxygen transfer into the pellets, as well as metabolites and toxic products release, become very limited or even nil (Forage et al., 1985; Brown et al., 1987). Under these conditions, the shear rate within the bioreactor varies extraordinarily, from the highest value close to the impeller to the lowest one next to the vessel wall, even in lab-scale stirred-tank reactors.

Fungal morphology in submerged cultures (pelleted vs. dispersed filamentous growth) is affected by a variety of cultural parameters like inoculum strength, medium composition, fermenter configuration (see below) and particularly, agitation rates (Papagianni, 2004; Fazenda et al., 2008). It was early suggested that a pelleted fungal morphology in *Sclerotium* sp. may produce higher β-glucan yields than diffuse mycelial cultures (Gibbs et al., 2000; Papagianni, 2004; Wucherpfennig et al., 2010; Schmid et al., 2011; Seviour et al., 2011a). Systematic studies on the relationship between aeration rate, mycelial morphology and EPS production were previously performed in *Cordyceps militaris*, finding that DO starvation at low airflow levels led to pellet autolysis and looser mycelial clumps. However, high aeration rates were neither beneficial. Intermediate values (2 vvm) led to compact mycelial pellets which resulted optimal for cell growth and EPS production (Park et al., 2002). In the case of *S. rolfsii* ATCC 201126, frequently working at 0.5 vvm and 400 rpm at fermenter scale, loose lenticular pellets are frequently associated to an optimal scleroglucan production (Valdez, 2013).

In stirred tank reactors (STRs), the average shear rate in the impeller zone is known to be a function of the impeller diameter and the impeller tip velocity. In pneumatic contactors such as bubble column and air-lift reactors (ALRs), it depends on the superficial gas velocity (Forage et al., 1985; Wecker and Onken, 1991). Bulk mixing may be improved by either increasing the impeller speed, increasing the gas velocity or altering the design of fermenter and/or impellers (Wang and McNeil, 1996).

A critical balance should be maintained concerning stirring rates, since scleroglucan would not be released from cell walls at sub-optimal shear stress. Low shear stress has been linked to the release of very low MW scleroglucan, whereas large biopolymer molecules remained adhered to mycelial walls (Rau et al., 1992). Poor bulk mixing may also add problems in terms of process monitoring and control (Wang and McNeil, 1996). However, very high stirring rates can usually lead to increased damage of hyphal cells along with the degradation of biopolymer macromolecular structure and the consequent reduction in broth viscosity (Nielsen, 1992; Rau et al., 1992).

Similarly, Schilling et al. (1999) confirmed that under high stirring rates, the produced scleroglucan showed a low MW as compared with that obtained after moderate agitation. A combination of moderate agitation plus high aeration rate allowed them to attain a maximum-MW scleroglucan. Although good agitation may be essential for mixing, if surpassed, it may adversely affect culture viability or physiology or even the EPS quality. Aeration, on the other hand, contributes to good mixing in a milder manner with no disturbance of the culture or polysaccharide molecular size (Giavasis et al., 2005). Additionally, foam control at high agitation rates requires special care since while some antifoams may considerably enhance EPS production in certain fungi, others (e.g., some vegetable oils) are known to be inhibitory (Stasinopoulos and Seviour, 1990; Fariña et al., 1998; Yang et al., 2000; Hsieh et al., 2006).

There is limited literature concerning the production of scleroglucan under different airflow and stirring rates. Rau et al. (1992) carried out batch cultivations with *S. glucanicum* CBS 52071 at different airflow rates using draft-tube propeller systems at a constant shear rate of 600 rpm. They reported that high airflow rates, in the order of 0.3 m^3^/h, were associated to larger growth rates and decreased scleroglucan formation.

##### Effect of other factors

Some additional effectors have been tested in order to increase scleroglucan biosynthesis. Among these, it is worth to mention the positive influence of high-osmotic-pressure conditions on scleroglucan biosynthesis. For instance, in *S. rolfsii* ATCC 201126, the influence of high osmolarity on scleroglucan production was partially evidenced by means of complementing the osmotic effects of culture media containing either 50 or 75 g/L-sucrose with additional NaCl or KCl, in order to mimic the 150 g/L-sucrose osmotic pressure (Fariña et al., 1998). Normally, in media with 150 g/L sucrose as C-source (e.g., MOPT medium), where EPS reaches the highest values, much of the sugar remains at the end of the batch. This finding was already associated to an EPS protective role and its increased production under high osmotic pressure environments, where scleroglucan might be part of an osmoregulation mechanism (Seviour et al., 1992; Fariña et al., 1998; Schmid et al., 2010).

In natural environments, EPS formation has been linked to substrate adhesion (e.g., biofilm formation) or desiccation prevention, among other roles (Donot et al., 2012). The production of EPSs has been also described as an adaptation strategy for microbial survival under stressing conditions such as freezing. Ice crystal formation, osmotic pressure variations, and water availability are factors normally related to cell stress during freezing. This would agree with the current use of polysaccharides (e.g., alginate) in cryopreservation techniques and would explain the protective properties of EPSs in Antarctic microbes to overcome lethal effects of freeze-thaw cycles, which are harsher than a continuously frozen environment (Selbmann et al., 2002b).

As it was noted in a previous report (Fariña et al., 1996), *S. rolfsii* ATCC 201126 was shown to be relatively halotolerant. Although osmosity exhibited a significant influence on β-glucan production, the highest EPS values reached with 150 g/L sucrose could not be equaled just by increasing culture medium osmotic pressure by means of salts addition. The osmotically active salts added to the medium did showed to increase EPS production but, the normal scleroglucan concentration obtained in 150-g/L-sucrose culture medium (MOPT) could not be achieved, probably because of the high ionic strength exerted by the NaCl or KCl surplus (Fariña et al., 1998). Similar evidences were found while examining the effects of osmotic pressure on the erythritol production by *Trigonopsis variabilis* (Kim et al., 1997).

Instead of salts, other non-metabolizable water activity (a_w_) adjusters like polyethylene glycol (PEG) 200 have been already cited in the literature, for example to study the osmotically modified enzymatic production in *Aspergillus niger* (Taragano et al., 1997) or to relate water activity depletion and the stimulation of EPS production in *Ganoderma lucidum* (Papinutti, 2010). In the latter case, and similarly to *S. rolfsii* ATCC 201126, an incomplete utilization of reducing sugars was found at high malt extract concentrations, suggesting a high C-source concentration as a positive effector for EPS production by decreasing water activity (Papinutti, 2010). Likewise, high solute concentrations in culture medium have been related to a reduced oxygen solubility and diffusion coefficient (Büchs, 2001), which may also stimulate EPS biosynthesis.

On the other hand, supplementation with sunflower oil and ascorbic acid, and particularly with L-threonine, led to a diminished EPS production in *S. rolfsii* ATCC 201126 (Fariña et al., 1998). Although some proteomic approaches already described the activation of heat-shock proteins and the increased expression of ATP citrate lyase (decreased TCA cycle activity) with increased EPS production by *Pleurotus tuber-regium* when stimulated by Tween 80 (Zhang et al., 2012), this supplementation to culture medium did not exerted stimulation toward EPS production in *S. rolfsii* ATCC 201126 (Fariña et al., 1998). In addition to the influence of culture medium composition, and with regard to external factors other than those already discussed, some authors also found that white or blue light facilitated glucan formation in *S. rolfsii* (Miller and Liberta, 1976), but no further information is currently available on this aspect.

**FIGURE 4:**
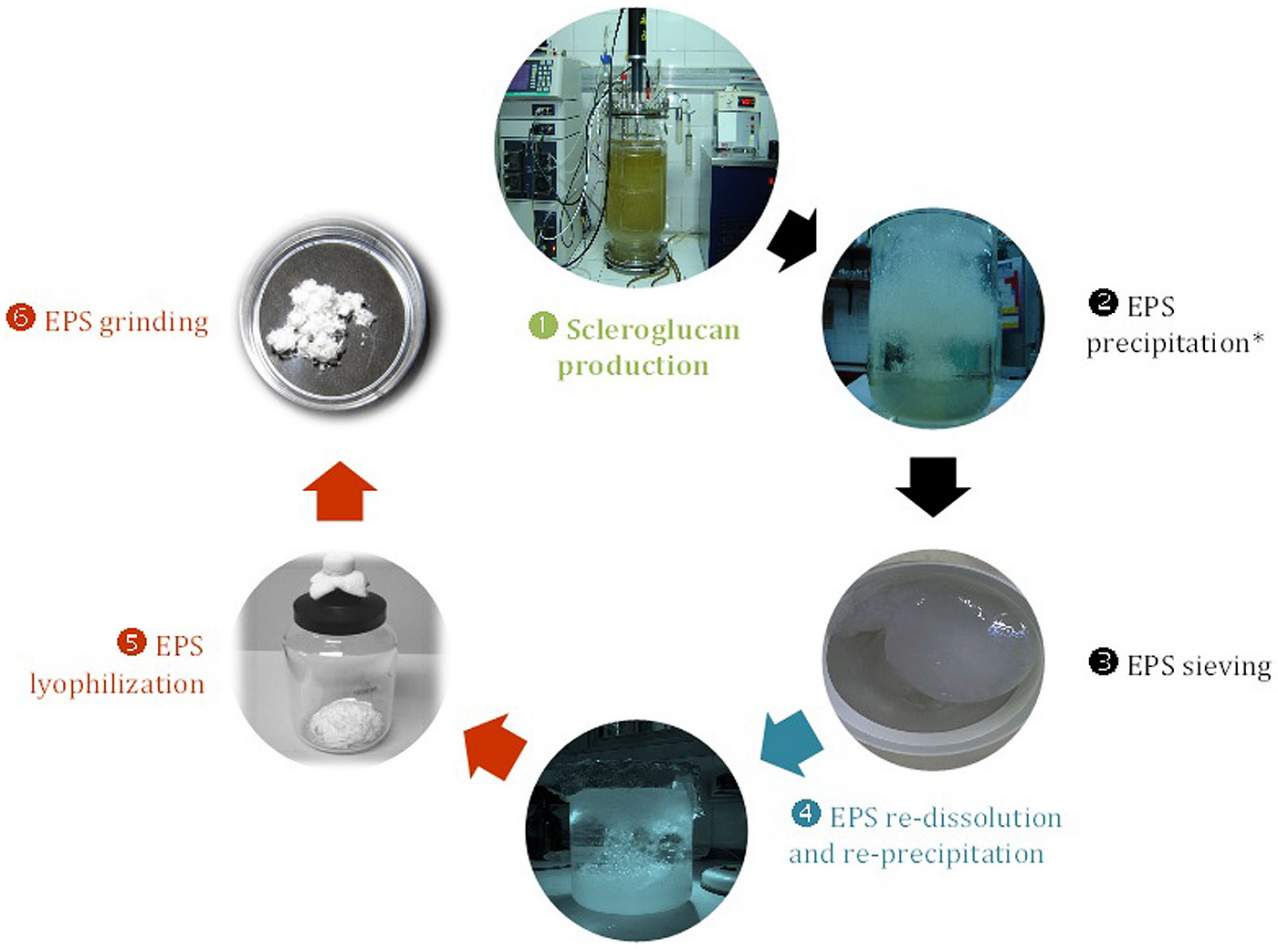
**Flowchart illustrating the main stages during production and downstream processing of scleroglucan from *S. rolfsii* ATCC 201126 (Fariña et al., 1998; Viñarta et al., 2013a).** Chain starts with 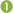
production at fermenter scale with MOPT culture medium under the following operative conditions: 400 rpm, 0.5 vvm and 30°C, in a BioFlo 110 fermenter (New Brunswick Sci.) with an 8 L-working volume. 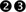
Preliminary EPS recovery; 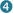
EPS purification; 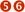
Final EPS treatments for storage and usage. 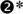
After biomass separation by centrifugation.

In the particular case of *S. rolfsii* ATCC 201126, several studies performed at shake-flask scale during the last decades allowed to establish optimal conditions for scleroglucan production (Fariña et al., 1996, 1998). Subsequently, when scaling-up to bioreactor scale, stirred-tank reactors were used under the following operative conditions: airflow rate, 0.5 vvm; stirrer speed, 400 rpm; temperature, 30°C and initial pH 4.5 (uncontrolled throughout the fermentation process). These conditions allowed to achieve a 1.5-fold increase in volumetric productivity (*Pr*, from 0.365 up to 0.542 g/L⋅h) in association to a significant shortening of the required cultivation time (from 72 to 48 h) and a final EPS concentration of 26 g/L (Fariña et al., 1998). Considering that typical batch cultures for scleroglucan production were usually described with a length of around 100 h (McNeil and Harvey, 1993), these latter results would be quite promising. A scheme depicting the optimized and standardized method for production and recovery of scleroglucan by *S. rolfsii* ATCC 201126 is shown in **Figure 4**.

Concerning scleroglucan production optimization, our first studies in this field were performed by using the one-factor-at-a-time method, which consists in changing one variable (nutrients, pH, temperature, etc.) while fixing the others at certain arbitrary levels (Fariña et al., 1998). This technique was also successfully applied to other EPSs (Xu et al., 2003). Nevertheless, because of the great number of factors that are involved in the process, this method usually implies a large number of experiments, and thus results very laborious and time consuming, and not always guarantees the disclosure of optimal conditions (Survase et al., 2006). Different methods have been proposed in the literature for the empirical modeling and optimization of EPS production which may behave more efficiently and/or accurately.

With reference to scleroglucan from *S. rolfsii* MTCC 2156, two main methods were proposed, one consisting in a statistical-based approach (response surface methodology) and the other one, in an artificial intelligence-based approach (artificial neural network-genetic algorithm; Desai et al., 2008). Both methods allowed a significant increase in scleroglucan titers (from 7.8 ± 0.54 g/L in non-optimized medium to 16.42 ± 0.68 g/L and 16.22 ± 0.44 g/L for artificial neural network-genetic algorithm and response surface methodology, respectively) with the requirement of a minimum number of experiments. Both methods provided a deep knowledge (e.g., interactions between different components) on the scleroglucan production system. Moreover, artificial neural network-genetic algorithm allowed predicting more accurate values of optimal conditions and optimum titers as compared to response surface methodology.

#### Fermenter Configuration

Relatively little information is available on the influence of fermenter configuration on α- or β-glucan yields in fungi (McNeil and Harvey, 1993; Gibbs and Seviour, 1996; Wang and McNeil, 1996). Bioreactor architecture is mainly involved in the efficient homogeneous mixing of the culture, especially promoting heat, oxygen and other substrates mass transfer to the cells (Rau et al., 1992; McNeil and Harvey, 1993). STRs are the workhorse in the fermentation industry, and they are the most utilized at both research and industrial scale (Lawford and Rousseau, 1989; Kang et al., 2000).

Two configurations are the most commonly used for fungal fermentations: the continuous STR and the ALR, whose different principles of mixing represent a high- and low-shear regime, respectively (Seviour et al., 1992; Gibbs et al., 2000; Papagianni, 2004). Even though data are available in the public domain, it is difficult to separate the complex individual effects of shear/mixing/mass transfer or DO levels and biomass morphology on β-glucan production, mainly, because of the performed experiments were not conceived to differentiate between each effect.

A stirrer system that imparts a high shear stress upon the medium normally uses Rushton turbine impellers, which pump out the medium radially from the turbine (McNeil and Harvey, 1993; Wang and McNeil, 1996; Gibbs et al., 2000; Papagianni, 2004; Fazenda et al., 2008). Radial flow (turbine) impellers are efficient at achieving oxygen transfer by virtue of their ability to increase turbulence. Their efficiency is, however, counteracted by the negative effect of this shear intensive system on the “quality” of the isolated exopolymer. Product quality is a relative term that can only be properly defined in terms of the end-use application (Lawford and Rousseau, 1991). As stirrer speed augments in a high-shear configuration, oxygen and heat mass transfer rates increase whilst the mixing times decrease (McNeil and Kristiansen, 1987). Fungal morphology is often quite different from that seen in low-shear systems (Gibbs et al., 2000; Papagianni, 2004; Fazenda et al., 2008; García-Ochoa and Gómez, 2009).

**FIGURE 5:**
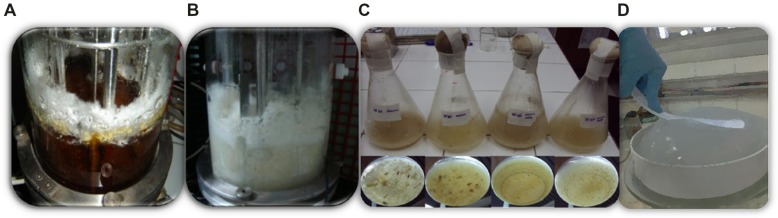
**Scleroglucan production by *S. rolfsii* ATCC 201126 with alternative C-sources: (A) Sugarcane molasses, (B) Corn starch or, (C) Ground lemon peel.** Aspect of EPS precipitate **(D)** after settling overnight at 5°C the corn starch-broth supernatant with an equivalent volume of ethanol 96°. **(A,B)** 2 L-working volume fermenter scale. **(C)** 100 mL-working volume shake-flask scale. Other culture medium components, as in PM_20_ (Fariña et al., 1998).

In smaller laboratory fermenters, and particularly for polysaccharide fermentations, wall effects become significant. It is not unusual to see impellers turning at high rpm and stagnant broth a few inches away. Poor mixing, particularly near the walls, is worsened by the presence of excessive baﬄing, cooling devices, pH and dissolved oxygen probes, and sampling lines (Wernau, 1985). These difficulties have been frequently observed during scleroglucan production by *S. rolfsii* ATCC 201126 (see below, **Figures 5A,B**).

The most common configuration to work under low-shear conditions is the ALR, which uses differences in hydrostatic pressure or density in order to achieve a fluid mixing. Air is injected through a sparger into the bottom of a riser tube, decreasing the effective density of the medium there. As bubbles rise to the top, they are released into the headspace; the medium becomes denser and it then descends to the vessel bottom *via* a downcomer or an external loop (McNeil and Harvey, 1993; Gibbs et al., 2000; Chisti and Jauregui-Haza, 2002; Papagianni, 2004; García-Ochoa and Gómez, 2009).

On the other hand, although less used, low-shear configurations in continuous STRs rely on modifications of the stirring systems and the impellers. Available low-shear impellers include axial flow and helical ribbon stirrers. The operation in both cases implies the pumping of the fluid from the top to the bottom of the fermenter at reduced liquid stress and against the airflow (Rau et al., 1992; McNeil and Harvey, 1993). For schizophyllan, a β-glucan similar to scleroglucan, EPS production reached higher values in fermenters with axial flow impellers than with helical ribbon stirrers (Rau et al., 1992).

The use of ALRs is being increasingly considered in fermentation industries instead of the traditional mechanically agitated bioreactor. Their design is mechanically simpler than the observed in STRs, and because of the absence of mechanical stirring, they are also less expensive to operate. Main advantages include low power inputs, relatively low shear, simple construction, and no moving mechanical parts, which additionally reduces contamination risks. Despite this, the comparatively low shear regime and lower oxygen transfer rates may represent difficulties at the time of cultivating filamentous fungi (Barker and Worgan, 1981; Blenk, 1985; Merchuk and Siegel, 1988; Allen and Robinson, 1989). These difficulties can be satisfactorily solved by the introduction of internal or external loops (Seviour et al., 2011b).

Higher EPS scleroglucan concentrations could be achieved with *S. glucanicum* NRRL 3006 (Wang and McNeil, 1995d) in a 120-L ALR with an external loop in comparison with the classical STRs, probably by satisfying a low oxygen demand when using the ALR fermenter architecture. Similarly, Kang et al. (2000), investigated scleroglucan production in an ALR with an internal loop. They found that scleroglucan productivity obtained in this system was comparable to those achieved in ALRs with an external loop or in stirred-tank reactors, presenting the additional advantage of low equipment investment and operational costs (Kang et al., 2000). Despite the clear economic advantages that ALRs offer for scleroglucan industrial or lab-scale production, these systems are not commonly used, perhaps because the lack of knowledge in this bioreactor configuration of both, process rheology and hydrodynamics (Wang and McNeil, 1996).

In conclusion, the technology of scleroglucan production still seems to be an aspect deserving further investigation, since the choice of fermenter design will help to determine the ultimate economic attractiveness of any proposed industrial process.

### Utilization of Alternative or Non-Conventional Substrates

At present and mainly for economic reasons, most of the commercialized polysaccharides are derived from plants, with the exception of xanthan and curdlan gums which are from bacterial origin (Clementi, 1997; Sutherland, 2008). The introduction of fungal EPSs like scleroglucan in the market is usually limited by low yields and high production costs. Therefore, unraveling these shortcomings may help to expand the scleroglucan market to relevant areas such as pharmaceuticals, cosmetics, food and agriculture (Singh et al., 1974; Pretus et al., 1991; McNeil and Harvey, 1993; Wang and McNeil, 1996). A tactic to reduce the costs and boost EPS production may involve developing fermentative processes that strategize the use of cheap and/or easily available substrates. Scleroglucan production commonly considers the use of conventional substrates like sucrose or glucose as C-source (Fariña et al., 1998; Survase et al., 2006; Schmid, 2008; Schmid et al., 2010), along with small amount of salts, yeast extract and nitrate as N-source. These latter are not quite expensive, however, their use might have more impact on production costs at industrial scale. Therefore, some authors have highlighted the importance of evaluating the employment of certain wastes or agro-industrial sub-products as potential scleroglucan production substrates.

Survase et al. (2007b) reported high scleroglucan production with *S. rolfsii* MTCC 2156 by using in culture medium either sugarcane molasses (∼19 g EPS/L) or coconut water (∼13 g/L). On the other hand, a reduction in production costs could also be achieved with *S. glucanicum* NRRL 3006 in a culture medium containing a sub-product coming from corn-based ethanol production [*Condensed Corn Soluble (CCS)*; Fosmer et al., 2010]. Similarly, other researchers found high scleroglucan yields by growing *S. glucanicum* NRRL 3006 in culture media based on starch-derived raw materials (Selbmann et al., 2002a). The use of alternative substrates may represent an attractive proposal not only from the economic point of view, but also at environmental level. It would allow reducing production costs at the same time of giving added value to non-valuable wastes or sub-products with scarce commercial value, what entails a revalorization of these alternative substrates. Additionally, their conversion into high-added-value bio products (EPS) would also contribute to the mitigation of the environmental impact generated by their eventual disposal in nature (Fernandes Silva et al., 2009; Taskin et al., 2011; Abdul Razack et al., 2013; Muhammadi and Afzal, 2014).

In recent years, with the aim of reducing production costs for scaling-up and based on the *S. rolfsii* ATCC 201126 ability to metabolize diverse C-sources, our group evaluated the possibility of using different available agro-industrial sub-products with relevance in the region (Valdez, 2013). Under-utilized biomasses such as vegetable and fruit processing residues constitute a promising source, being generated in huge amounts every year and representing an environmental and economic problem of worldwide concern. Among these residues, peels, seeds and pulps constitute a 30–50% of input materials contributing with high organic matter to the environment. These agro-wastes might then be envisaged as suitable C-sources for revalorization through the production of high added-value biotechnological products (“biorefinery” strategy) such as EPSs (Poli et al., 2011). Simultaneously, this would aid to cope with the depletion of natural resources and environmental concerns.

Among alternative substrates, the use of sugarcane molasses and lemon peel have been tested for scleroglucan production by *S. rolfsii* ATCC 201126 (Valdez, 2013; Montes de Oca, 2014). The former one is a liquid by-product of the sugar industry with a dark brown color, highly viscous and homogeneous appearance, where the major sugar component is sucrose. Some ions like Mg^2+^, Mn^2+^, Al^3+^, Fe^3+^, and Zn^2+^ may also be present in variable proportions (Prescott and Dunn, 1987).

Molasses (≡20 g/L reducing sugars) with no pre-treatment could be used in culture medium which led to proper growth and scleroglucan production in *S. rolfsii* ATCC 201126 (Valdez, 2013). Conversely, Roukas and Liakopoulou-Kyriakides (1999) proposed a pre-treatment of this substrate prior to pullulan production, due to the presence of heavy metals with inhibitory effects on microbial growth and EPS-linked enzymatic activities. In our case, the maximal scleroglucan production at bioreactor scale with molasses as C-source (5.11 g/L; **Figure 5A**) was moderately lower than the one normally achieved in control medium with 20 g/L sucrose (6.87 g/L). The crude EPS obtained under these conditions exhibited an undesirable brownish coloration, likely related to the presence of melanoidins as the main pigment in sugarcane molasses (Chandra et al., 2008). However, this pigmentation could be significantly reduced once EPS downstream processing was completed, thus reflecting a higher purity grade.

As the quality and EPS structural features may vary depending on the C-source, the obtained EPS was evaluated on its purity degree and rheological characteristics (Castillo et al., 2014). The EPS coming from molasses medium showed high purity values (93% w/w total sugars, 9.1% w/w reducing sugars, with minimal protein contamination). Aqueous solutions containing this EPS showed non-Newtonian pseudoplastic behavior with better rheological properties (higher consistency coefficient, *K*, and lower flow behavior index, *n*) as compared to the EPS obtained from conventional sucrose, at identical EPS concentration.

These results allowed us to confirm the feasibility to produce high-quality scleroglucan from sugarcane molasses, which may represent a more economic option, especially for regions where this sugar industry by-product is abundant (Koller et al., 2012). That would be valuable at industrial level at the time of facing large-scale production and taking into account the wide potential uses of this EPS. Applications may include from the utilization of the whole culture broth, as for enhanced oil recovery (EOR; Sandford, 1979; Holzwarth, 1984), to the use of a refined grade EPS (e.g., for cosmetic, pharmaceutical or medical applications; Pretus et al., 1991; Falch et al., 2000; Mueller et al., 2000; Coviello et al., 2005; Laroche and Michaud, 2007; Viñarta et al., 2007; Giavasis, 2014).

The use of commercial corn starch (≡20 g/L reducing sugars) has also been tested as C-source for scleroglucan production by *S. rolfsii* ATCC 201126 at bioreactor scale (Valdez, 2013). Under these conditions, EPS production (7.95 g/L; **Figure 5B**) exceeded the obtained values with the same concentration of either sucrose or molasses as C-source. The obtained EPS was more easy to precipitate (**Figure 5D**) and the efficiency of the recovery process thus ascended from the typical ∼30% (28.5% with sucrose and 33.2% with molasses, respectively) to above 50%. Purity analyses of EPS revealed similarities in total and reducing sugars, and protein contents as in the EPSs obtained with sucrose or molasses. Rheological properties, however, for an equal EPS concentration, indicated a lower *K* value in comparison to the molasses-EPS but higher than the traditional sucrose-EPS. In this sense, our results have oriented us to the study on the use of low-cost amylaceous materials or residues, as potato washing wastewater. The alternative use of cheese whey (Fernandes Silva et al., 2009) and olive mill wastes (Dermeche et al., 2013) is also being considered.

Another C-source more recently tested with *S. rolfsii* ATCC 201126 was the ground- and acid-treated lemon peel obtained from a local citrus-processing factory (Montes de Oca, 2014). The use of this substrate (≅15 g/L reducing sugars; **Figure 5C**) led to interesting EPS production values (∼13 g/L), even surpassing the final EPS concentrations (5–8 g/L) in sucrose-, molasses-, or corn starch-containing media (Valdez, 2013). However, the maximal EPS concentration in pre-treated citrus-peel-containing media could just be achieved at 7 days of cultivation instead of the 2–3 days required for culture media with sucrose, molasses or corn starch as C-sources. An additional aspect to consider is that pectin (released from citrus peel to culture broth) may co-precipitate along with EPS when alcohol is added for the recovery process. This hitch could be partially solved by previously treating the samples (48 h at 40°C) with commercial pectinase (Montes de Oca, 2014). The practicability of using citrus peel for EPS production should then be carefully weighed taking into account that factors such as the pre-treatment of raw material, the longer cultivation time and the remaining pectin interference may deleteriously influence the production process costs. On the other hand, despite the cited limitations for EPS production, the finding of an appropriate fungal growth along with the secretion of a wide variety of enzymes with this substrate would allow to suggest these cultivation conditions for the production of other biotechnologically relevant products, such as hydrolytic and ligninolytic enzymes (Montes de Oca, 2014).

As a final point, it is already described in the literature that *S. rolfsii* and *S. glucanicum* behave as phytopathogens with the natural ability to produce a wide enzymatic spectrum including cellulases, phosphatases, arabinases, exogalacturonases, polygalaturonases, galactosidases, and exomannanases (Survase et al., 2007b). It can be therefore expected that some plant residues such as sugarcane bagasse (Koller et al., 2012), solid olive-mill by-product called “alperujo” or olive mill wastewater (“alpechin”; Dermeche et al., 2013) may also be evaluated as potential unconventional raw materials for the low-cost scleroglucan production within an eco-sustainable framework. Nevertheless, in spite of all the work performed up to date and their prospects, scleroglucan production at industrial scale still remains limited to glucose or sucrose, which makes the finding of alternative substrates as well as the reduction of costs a continuing challenge to keep working on.

## Downstream Processing

As relevant as improved cultivation conditions are for achieving high polymer yields, the downstream processing also represents a crucial step in order to ensure a high recovery yield of a refined-grade polysaccharide. This stage may affect critical macromolecular features such as polymer MW, DP, DB, purity and composition, and also constitutes a significant part of the total production costs (Wasser, 2002; Wang et al., 2010; Viñarta et al., 2013a,b). This processing stage optimization becomes imperative and, depending on the desired purity or the intended use (which usually requires given physico-chemical properties), different extraction and purification techniques should be developed.

In the case of extracellular slimy polysaccharides, this process generally involves the sterilization or pasteurization of the fermentation broth in order to kill microbial cells, inactivate undesirable enzymes (e.g., glucanases) and facilitate EPS detachment from cells. The subsequent removal of biomass is frequently carried out by filtration or centrifugation. The polysaccharide in the cell-free filtrate or centrifugate is then precipitated with alcohol, followed by further purification steps (if required) such as ultrafiltration, gel permeation/ion-exchange chromatography, or diafiltration. The end product is finally obtained after drying with either air/inert gas under vacuum, spray drying, or lyophilization, plus a final milling to the desired mesh size (Giavasis, 2013).

Referring to the *S. rolfsii* ATCC 201126 scleroglucan downstream processing, different strategies were evaluated in order to finally establish an optimized purification protocol. That led to an industrially acceptable recovery of EPS with high purity grade (∼98%; Fariña et al., 2001; Viñarta et al., 2013b). This protocol commonly involves a step of homogenization to facilitate the EPS release from mycelium and to weaken the intermolecular polysaccharide association, so that it is completely dispersible in water. This is followed by a threefold dilution of culture broth with distilled water, which otherwise is extremely viscous and difficult to process, and a final neutralization with NaOH. Thereafter, the diluted/neutralized broth is heated (80°C for 30 min) to inactivate eventually produced glucan-degrading enzymes and to enhance glucan solubilization in water, and finally centrifuged (27500 × *g*, 15°C, 20 min). The EPS from clear supernatant is then precipitated by adding an equivalent volume of an organic solvent, commonly a lower alcohol.

**FIGURE 6:**
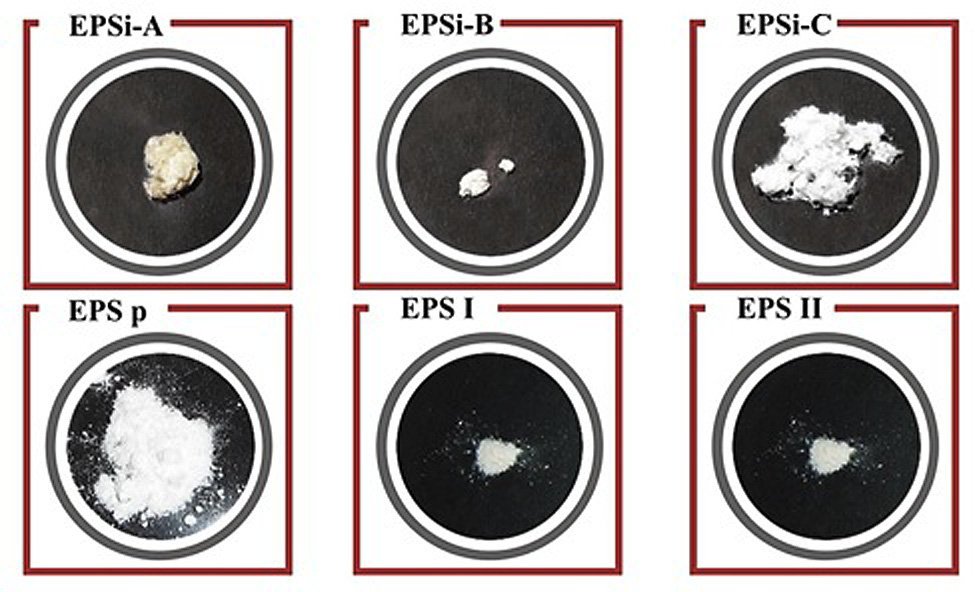
**Appearance of EPSs produced by *S. rolfsii* ATCC 201126 at fermenter scale in MOPT culture medium and subsequently downstream processed.** EPSi corresponds to scleroglucan precipitated with isopropanol after 1 (A), 2 (B), or 3 (C) re-dissolution/re-precipitation steps. EPSp illustrates the EPS precipitated with PEG. EPS I and EPS II correspond to ethanol-precipitated scleroglucans after 48 or 72 h of cultivation (Fariña et al., 1998; Viñarta et al., 2013b).

Generally speaking, when a polysaccharide is present, fungal mycelia are easier to remove from diluted broths; but the additional cost of re-concentration normally imposes an economic drawback (Wernau, 1985). The possibility to achieve a high recovery of pure scleroglucan from diluted supernatants of *S. rolfsii* ATCC 201126, from ∼30% (with sucrose or molasses media) up to 50% (with corn starch medium), attenuates this disadvantage. Concerning the precipitation step for EPS recovery, different alcohols (ethanol 96°, isopropanol, and PEG) were tested (Johal, 1991; Fariña, 1997; Fariña et al., 2001; Viñarta et al., 2013b). Among them, we found that ethanol 96° and isopropanol allowed obtaining the highest recovery, high purity degree, finest appearance, optimal water solubility and remarkable rheological properties of EPS (**Figure 6**). In addition to the first precipitation step at the end of centrifugation, the inclusion of a three-step re-precipitation/re-dissolution cycle (**Figure 4**) with either ethanol or isopropanol, was the best methodology to achieve a refined-grade scleroglucan, suitable for example for biomedical testing (Fariña et al., 2001; Viñarta et al., 2013b).

## Operational Strategies

As scleroglucan represents a growth-associated metabolite, it has been early recognized that those conditions favoring growth will also do so on EPS production. However, it was noted that some required conditions that seem to promote polysaccharide production do not always imply the stimulation of growth but the opposite. This gave place to bi-staged processes, where optimal conditions for biomass production are supplied during the first stage and then, once a critical biomass has been produced, the conditions are changed in order to stimulate EPS production (Sutherland, 1982).

The commonly selected method for EPS production is the *batch process*, where the producing microorganism is inoculated into the culture medium which contains all the required nutrients. Under these conditions, growth and EPS production take place until the exhaustion of a given critical substrate (which usually turns limiting). On the other hand, the *continuous culture* strategy, which consists in the uninterrupted addition of fresh culture medium whilst spent broth (containing part of the biomass and the product of interest) is simultaneously harvested, is not frequently used for EPS production (Rosalam and England, 2006). Nevertheless, this latter methodology has been eventually employed to study biochemical and physiological aspects related to some polysaccharide-production processes (Sutherland, 1982). In the case of scleroglucan, an alternative continuous culture process at lab and industrial-scale has been patented years ago (Maier, 2004).

Unfortunately, in contrast to batch-wise cultivation, continuous culture resulted not feasible under non-aseptic conditions, being less effective with regard to yield and product quality, as compared to batch cultures (Schilling, 2000). It may also be worthwhile to highlight that for some microorganisms such as the xanthan-producer *Xanthomonas campestris*, the continuous culture strategy might lead to the undesirable selection of poorly EPS-producing strains (Sandford, 1979).

In order to improve scleroglucan production by *S. glucanicum* NRRL 3006, some researchers developed a *bi-staged process*. During the first phase of cultivation, pH was controlled at 3.5 with the aim of promoting optimal growth, and thereafter pH was raised up to 4.5 to favor polysaccharide biosynthesis. The second stage allowed achieving a 10% reduction of by-product (oxalic acid) formation, simultaneously with an increased scleroglucan concentration. This fact may reflect that pH levels (i.e., 4.5) higher than those for optimal growth prompt the carbon flux toward biopolymer synthesis (Wang and McNeil, 1995c). In a similar way, the chosen process temperature is often a compromise between the optimal temperature for growth and the one for EPS production. A bi-staged process of temperature could also be adopted for the improvement of polysaccharide synthesis at a second stage (Wu et al., 2010). The use of dual-stage production processes have been also successfully applied to other microbial polysaccharides in order to achieve different optimal conditions, either for growth or biopolymer synthesis (Zheng et al., 2013).

Although batch cultures are usually adopted on an industrial scale, a *fed-batch process* with a stepwise addition of the C-source (and further nutrients) may often improve the final product concentration, thus eliminating any eventual substrate inhibition. The feeding of concentrated medium to the culture is commonly performed at a rate that prevents the carbon source from reaching the threshold value for catabolite repression (Spohr et al., 1998). In the case of scleroglucan from *S. glucanicum* NRRL 3006, this methodology has been reported to avoid the inhibitory effects of high (≥45 g/L) initial sucrose concentrations (Taurhesia and McNeil, 1994b). Supplemented batch cultures (at around 72 h) with additional sucrose after the initial growth phase overcame these difficulties while improving EPS production and its yield on C-source. The feeding strategy has also been reported for polysaccharides such as curdlan (Lee et al., 1997), gellan (Wang et al., 2006), scleroglucan (Survase et al., 2007a; Fosmer et al., 2010), ganoderan (Tang and Zhong, 2002), and *Saccharomyces cerevisiae* glucan (Kim and Yun, 2006).

## Metabolic and Genetic Engineering as a Tool for Increasing EPS Production

A better understanding of the EPS biosynthesis regulation will be crucial to face a rational and not empirical optimization of polysaccharide production. Acquiring this information might be challenging, but essential to produce tailor-made EPSs with enhanced bioactivity and more attractive physicochemical properties (Angelova and Hunkeler, 1999). The advancement in practical tools for genetic manipulation of fungi would be the main ally for elucidating EPS biosynthetic pathways and their regulation (Seviour et al., 2011a).

At the moment, much is known about the physicochemical properties of scleroglucan and its applications. Conversely, up to a few years ago, there was a lack of information regarding its biosynthetic pathway and regulation, at both genetic and enzymatic levels. First discernments in this field were recently published by Schmid et al. (2010) who established the first sequence database for *S. rolfsii* ATCC 15205. These authors compared the gene expression and transcriptomes of *S. rolfsii* under conditions of either maximum or minimum scleroglucan production. Obtained data allowed them to predict the pathways for scleroglucan and oxalate synthesis and degradation, and led to the knowledge that metabolic pathways for scleroglucan and oxalate synthesis were not coupled to each other, as it was believed, but oxalate synthesis may be rather linked to biomass formation (Schmid et al., 2011).

The same group of researchers could also identify important unigenes putatively involved in determining scleroglucan yields, and found that almost all the genes supposed to be involved in scleroglucan synthesis, glycolysis, TCA, and glyoxylate cycles were not differentially transcribed under high- or low scleroglucan producing conditions. Their results further suggested that the regulation of polymer synthesis would be rather linked to mechanisms ensuring fungal survival under anoxic and other stress conditions (Schmid et al., 2011). The complete genome sequencing of scleroglucan-producing strains, along with metabolomics and proteomics inputs, would provide a real boost toward the optimization of scleroglucan production.

## Concluding Remarks

With the advent of modern biotechnology, a new perspective for the use of fungi as generators of innovative products was completely opened up. Scleroglucan biopolymer belongs to these original products of fungal origin, and due to its versatility and unique properties it may find its way into numerous industries, such as oil, cosmetics, food, and pharmaceuticals. Microbial production of scleroglucan, both at lab and industrial scale, remains as one of the most multifaceted processes currently known. The optimization of scleroglucan production as well as solving its related obstacles will ensure the economic success of this development and its competitiveness. That will involve integrating the inputs from multiple disciplines such as microbiology, biochemical engineering, process engineering, statistics and genetics.

In this chapter we have reviewed much of the knowledge on scleroglucan production to date, particularly regarding the process design and optimization, and by examining both the available literature and our experience. There are still several hitches mainly regarding to the bioreactor design and process development for large-scale scleroglucan production, which are expected to be solved during the following years. Additionally, a deeper insight into the scleroglucan biosynthesis and its regulatory networks will also be crucial for the process optimization. This information, together with genetic engineering and transformation techniques, may allow modifying the expression of scleroglucan-biosynthesis related genes in order to divert more carbon flux toward polysaccharide production.

## Author Contributions

JF conceived the idea and established the general outlines. NC wrote the table draft and a major part of the manuscript. AV wrote a sub-section of the manuscript. JF prepared illustration panels and critically revised the intellectual content of the work. All authors read and approved the final version of the manuscript.

## Conflict of Interest Statement

The authors declare that the research was conducted in the absence of any commercial or financial relationships that could be construed as a potential conflict of interest.
